# Tadalafil-Induced Generalized Bullous Fixed Drug Eruption: A Rare Side Effect of a Common Drug

**DOI:** 10.7759/cureus.39649

**Published:** 2023-05-29

**Authors:** Devansi Sarawgi, Olympia Rudra

**Affiliations:** 1 Dermatology, Institute of Post Graduate Medical Education & Research, Kolkata, IND

**Keywords:** phosphodiesterase-5 inhbitors, adverse effects, rare side effect, fixed drug eruption, tadalafil

## Abstract

Tadalafil is a phosphodiesterase-5 (PDE-5) inhibitor, an FDA-approved treatment for erectile dysfunction (ED), pulmonary arterial hypertension (PAH), benign prostate hyperplasia, etc. It is also widely used by otherwise healthy individuals for recreational purposes. Fixed drug eruption (FDE) is a distinctive type of adverse drug reaction in which every exposure to the offending medication leads to the appearance of lesions at the same ‘fixed’ sites. A sharply defined erythematous patch or plaque with a violaceous hue is typically seen. A clinical variant featuring classic FDE lesions along with blistering in at least three out of six anatomical sites or involving at least 10% of body surface area is known as generalized bullous fixed drug eruption (GBFDE). Tadalafil-induced FDE is in itself an uncommon phenomenon, with only a handful of documented cases, none of which seem to have reported GBFDE-type presentation post-tadalafil intake. Here, we present a case of GBFDE following tadalafil administration.

## Introduction

Tadalafil, like sildenafil and vardenafil, is a phosphodiesterase-5 (PDE-5) inhibitor. Its therapeutic usage, as approved by FDA, includes erectile dysfunction (ED), pulmonary arterial hypertension (PAH), and benign prostate hyperplasia [1]. It is also widely used by otherwise healthy individuals for recreational purposes [2]. Headache, dyspepsia, back pain, nasal congestion, etc., are some of its well-known side effects [3]. Fixed drug eruption (FDE) is a distinctive mucocutaneous adverse effect caused by the intake of certain drugs in a susceptible individual [2]. Lesions of FDE characteristically appear at the same ‘fixed’ locations every time the offending medication is used [1]. Round to oval, well-defined erythematous patches or plaque with a violaceous hue is the usual presentation. Few blisters may sometime be present over the lesions. Generalized bullous fixed drug eruption (GBFDE) is an uncommon form that may be mistaken for other severe drug reactions like Steven-Johnson syndrome or toxic epidermal necrolysis [4]. Tadalafil-induced FDE is a rare phenomenon, with only a handful of cases documented in the literature, none of which to date have reported a GBFDE after tadalafil intake [2]. Here, we present a case of GBFDE in a patient post-medicating with tadalafil.

## Case presentation

A 46-year-old married man presented to our OPD with multiple itchy, mildly painful, erythematous patches with blisters of varying sizes over his body for the last three days. It was not associated with fever, malaise, or other systemic features. The patient gave a history of two similar events of smaller magnitude; one five months back with few hyperpigmented patches over lips, chest, and upper limb while two months back, similar lesions over the same sites had appeared along with few vesicles over his back and buttock. In both previous episodes, lesions had disappeared on their own over a few days, hence patient didn’t seek medical consultation. 

**Figure 1:**
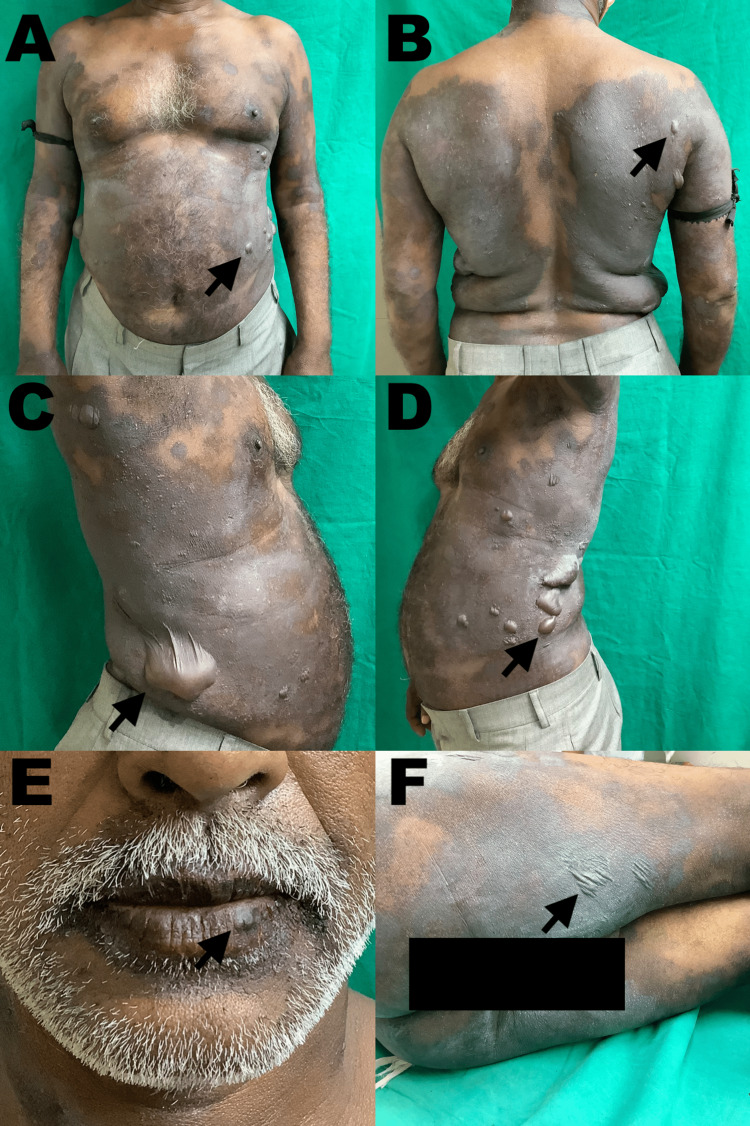
Cutaneous manifestations at presentation Multiple well-defined hyperpigmented patches with overlying flaccid blisters (black arrows) over the abdomen (A), back (B), right flank (C), left flank(D), lower lip (E), and right posterior thigh(F)

On examination, multiple well-defined hyperpigmented patches with violaceous hue were present symmetrically over the chest, back, upper and lower extremities, buttock, lips, and genitalia. Multiple vesiculobullous lesions with sizes varying from 0.3 cm to 8 cm were noted over the flanks, chest, back, arms, thighs, and lips (Figure 1).

**Figure 2:**
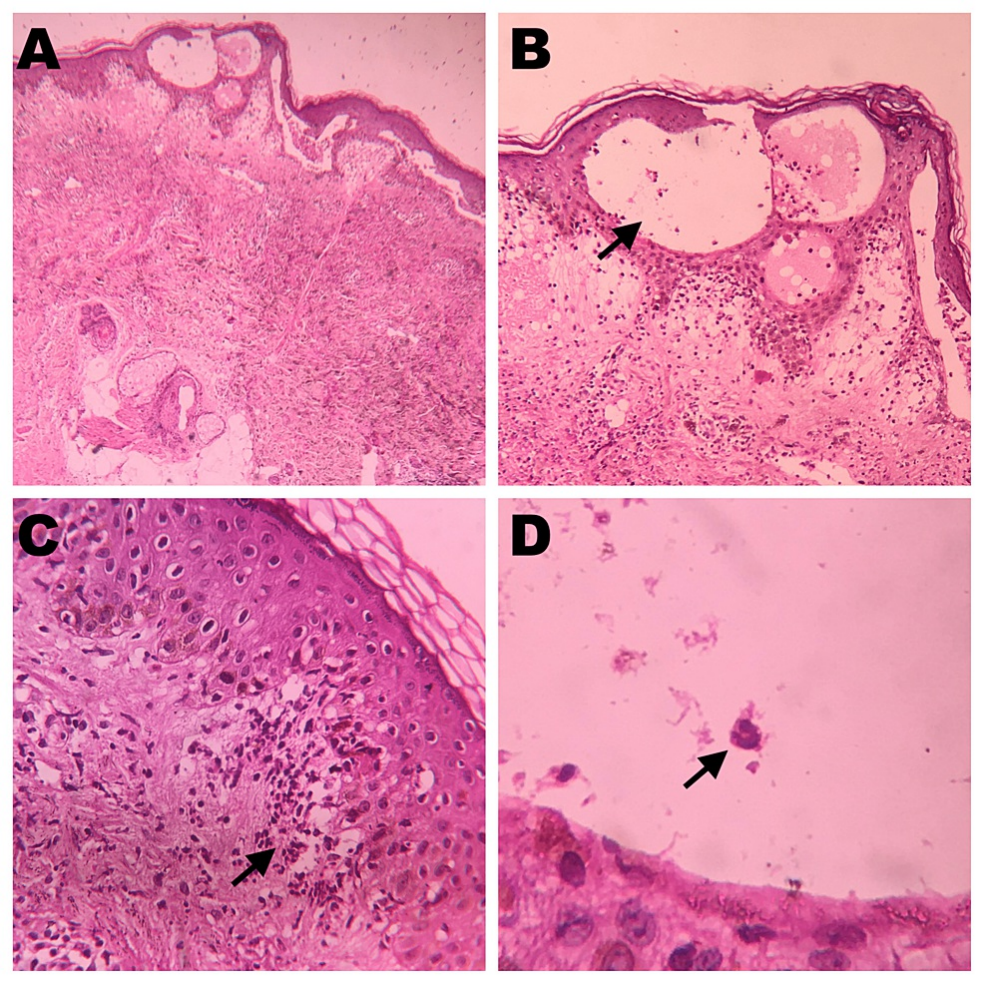
Histopathological examination Seen are an intraepidermal bulla (A) at 4x magnification, hydropic degeneration of basal layer (B) at 10x magnification, with eosinophilic infiltration (C) at 40x magnification, in hematoxylin and eosin stain (D).

Systemic examination was non-contributory. Routine lab investigations were found to be within normal limits. On histopathology, intraepidermal bulla with hydropic degeneration of the basal layer and lymphoeosinophilic infiltrates were noted (Figure 2).

**Figure 3:**
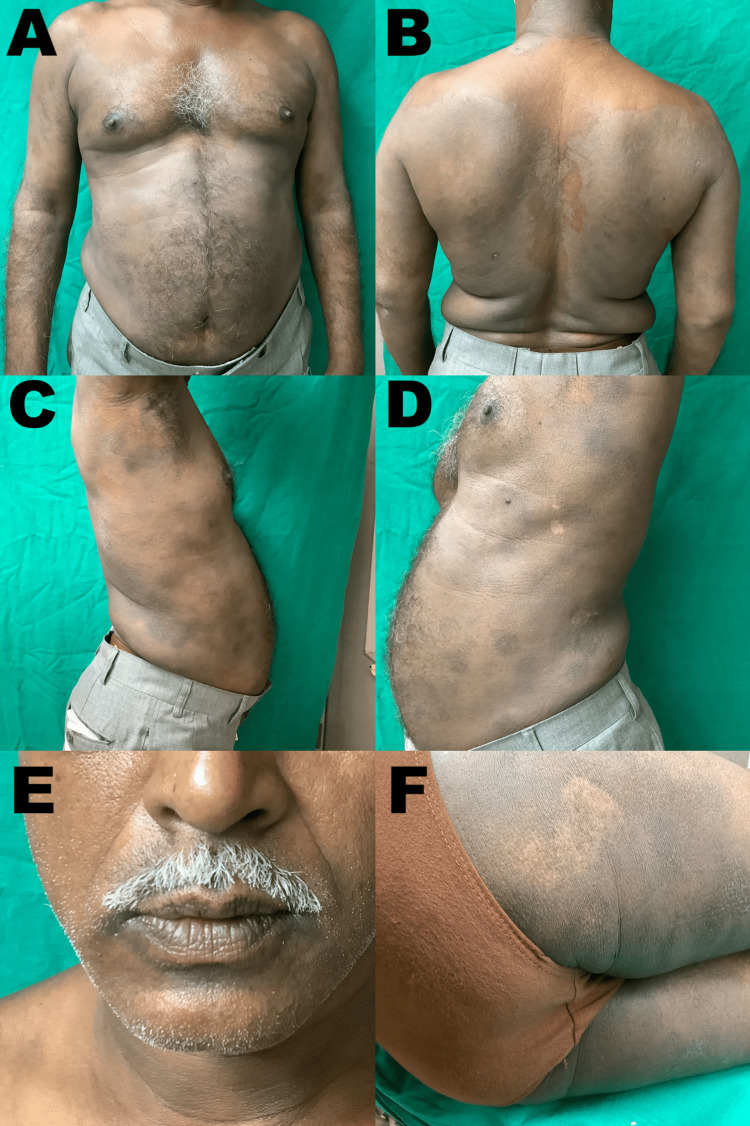
Post-treatment follow up Resolution of lesions with mild postinflammatory hyperpigmentation after 10 days of oral prednisolone (40 mg/day) therapy

Based on history, clinical features, and histopathological findings, we diagnosed it as a case of GBFDE. The patient reported that he had no comorbidities and was not under any medication. He also denied any form of self-medication at first but eventually recalled having taken an over-the-counter (OTC) drug for recreational purposes a few days prior to the appearance of all three episodes. Aspiration of large bullous was done, and prednisolone (40 mg) once daily was started. As advised, the patient brought the ‘offending’ drug on his next OPD visit, which turned out to be a tablet of tadalafil (5 mg). On calculating the Naranjo adverse drug reaction probability scale, the score was 8, thereby making tadalafil the probable cause of the FDE. The patient was properly counseled regarding avoidance of tadalafil or similar drug usage in the future. Within 10 days of starting therapy, all lesions resolved with mild postinflammatory hyperpigmentation (Figure 3).

## Discussion

Fixed drug eruption is the most frequently occurring cutaneous drug reaction in India [5]. The presentation can be varied and includes pigmentary or non-pigmentary lesions, generalized involvement with or without bullous eruption, or even erythema multiforme (EM)-like features [1]. Genitalia and extremities are the commonly affected sites, though any mucocutaneous area can be involved [1,5].

Pathogenesis is not well known, but it is considered to be CD8+ mediated type 4 hypersensitivity reaction to certain drugs or their metabolites [2]. Tetracyclines, NSAIDs, sulphonamides, etc., are well-known culprit drugs [4]. The reappearance of lesions at the same sites on re-administration of the offending agent is its most unique feature [1]. However, with every subsequent exposure, severity, and area of involvement may increase, and even generalized eruption may occur [4].

Generalized bullous fixed drug eruption is an unusual form of FDE where along with typical FDE lesions, the vesiculobullous eruption is seen in at least three of the six anatomical sites i.e., head and neck, front of the trunk, back, upper and lower limbs, and genitalia; or at least 10% of the body surface area [4]. In our case, blistering was noted over four anatomical regions in addition to the usual FDE eruptions, thereby fulfilling the above criteria for GBFDE. 

Fixed drug eruption lesions typically tend to fade spontaneously without scarring on withdrawal of the causative medication [4]. The immediate stoppage of the culprit drug, along with topical corticosteroid application and oral antihistamines, is sufficient in most cases, while oral corticosteroids may be required in disseminated forms [1]. Counseling the patient regarding avoidance of such drugs is also imperative for preventing recurrence in the future [5]. 

Tadalafil is a current generation PDE-5 inhibitor with more flexible dosing and steady pharmacological action. It leads to vascular smooth muscle relaxation and increases blood flow to the corpus cavernosum thereby helping obtain and sustain an erection [3]. There seems to be a surge in its demand, both for medical indications like ED and PAH as well as for recreational activities [1]. Enticing advertisements promising better sexual satisfaction upon its usage and easy availability over the counter have contributed to its injudicious use by the general public [2]. However, social taboo regarding the use of such ‘sexual stimulant’ drugs often prevents its consumer from seeking treatment in case of any detrimental reaction following its intake. Hence monitoring the side effects of these drugs is a difficult task, and its less common adverse reaction may be underreported [1]. Only a few cases of FDE due to tadalafil has been known till now [1,2].

## Conclusions

After an extensive literature review, no case of tadalafil-induced GBFDE was found, and to the best of our knowledge, this is the first case of GBFDE caused by tadalafil. Through this case, we want to highlight this supposedly rare but possibly fatal side-effect of tadalafil, a commonly misused OTC drug.

## References

[1] Chiu AW, Stenstrom R (2018). A case report of tadalafil-associated fixed drug eruption. J Pharm Pract.

[2] Das S, Das S, Chowdhury J, Bhanja DC (2014). Non pigmenting mucosal fixed drug eruption due to tadalafil: a report of two cases. Indian Dermatol Online J.

[3] Frajese GV, Pozzi F, Frajese G (2006). Tadalafil in the treatment of erectile dysfunction; an overview of the clinical evidence. Clin Interv Aging.

[4] Podder I, Chandra S, Das A, Gharami RC (2016). Doxycycline-induced generalized bullous fixed drug eruption. Indian J Dermatol.

[5] Ghoshal L, Sinha M (2015). Fixed drug eruptions with modafinil. Indian J Pharmacol.

